# A New Look at Structural Changes in the Aortic Root in Aortic Valve Stenosis

**DOI:** 10.17691/stm2022.14.2.05

**Published:** 2022-03-28

**Authors:** E. Kobelev, T.A. Bergen, A.R. Tarkova, O.V. Krestyaninov, E.E. Bobrikova, I.K. Safro, A.M. Chernyavsky, I.Yu. Zhuravleva

**Affiliations:** Junior Researcher, Research Department of Radiation and Instrumental Diagnostics; Meshalkin National Medical Research Center of the Ministry of Health of the Russian Federation, 15 Rechkunovskaya St., Novosibirsk, 630055, Russia;; Head of the Research Department of Radiation and Instrumental Diagnostics; Meshalkin National Medical Research Center of the Ministry of Health of the Russian Federation, 15 Rechkunovskaya St., Novosibirsk, 630055, Russia;; Leading Researcher, Research Department of Radiation and Instrumental Diagnostics; Meshalkin National Medical Research Center of the Ministry of Health of the Russian Federation, 15 Rechkunovskaya St., Novosibirsk, 630055, Russia;; Head of the Research Department of Endovascular Surgery, Research Institute of Blood Circulation Pathology; Meshalkin National Medical Research Center of the Ministry of Health of the Russian Federation, 15 Rechkunovskaya St., Novosibirsk, 630055, Russia;; Researcher, Research Department of Radiation and Instrumental Diagnostics; Meshalkin National Medical Research Center of the Ministry of Health of the Russian Federation, 15 Rechkunovskaya St., Novosibirsk, 630055, Russia;; Student, Medical Faculty; Novosibirsk National Research State University, 1 Pirogova St., Novosibirsk, 630090, Russia; Professor, General Director; Meshalkin National Medical Research Center of the Ministry of Health of the Russian Federation, 15 Rechkunovskaya St., Novosibirsk, 630055, Russia;; Professor, Director of the Institute of Experimental Biology and Medicine; Meshalkin National Medical Research Center of the Ministry of Health of the Russian Federation, 15 Rechkunovskaya St., Novosibirsk, 630055, Russia;

**Keywords:** aortic stenosis, aortic valve, transcatheter aortic valve implantation, computed tomography, 3D modeling of the aortic root

## Abstract

**The aim of the study** was to identify new anatomical landmarks of the aortic root and the relationship between the sizes of anatomical structures using the method of computed tomography angiography to improve models of heart valves and the methods for their selection in clinical practice.

**Materials and Methods:**

The dataset of computed tomography angiography prior to aortic valve replacement in 262 patients was analyzed. The mean age was 75.0±5.9 years. 99 (37.8±3.0%) men and 163 (62.2±3.0%) women took part in the study. The annulus fibrosus, sinotubular junction, and height of the sinuses of Valsalva were measured.

**Results:**

In the tricuspid aortic valve group (n=251), in more than 50% of the cases, the diameter of the annulus fibrosus ranged from 23 to 26 mm. No significant association between the diameter of the annulus fibrosus and patient height (r=0.35; p=0.01) or body surface area (r=0.25; p=0.01) and the height of the sinuses of Valsalva (r=0.34; p=0.01) were revealed. Based on the ratio of the height of the sinuses of Valsalva and the diameter of the annulus fibrosus, three variants of the structure of the aortic root were identified: type A — *K*>1.05; type B— 0.95≤*K*≤1.05; type C— *K*<0.95. Type C of the aortic root was found to predominate in most cases, namely, in 98.0±0.9% (n=246).

In the bicuspid aortic valve group (n=11), 2 patients had a type A of the aortic root, 1 patient had a type B, and 8 patients had a type C.

**Conclusion:**

A classification of variants of the aortic root structure has been proposed, which will be useful not only for practitioners when choosing a treatment method, but also for researchers to understand the structural characteristics of the aortic root in patients with its pathology.

## Introduction

Aortic valve stenosis is common in the aging population and often requires surgical treatment [1]. Over the past decades, transcatheter aortic valve implantation (TAVI) has been actively introduced into surgical practice, which has evolved, over time, from a very complex and dangerous operation into a routine procedure [2]. The improvement of minimally invasive treatment methods and the expansion of indications for TAVI in moderate and asymptomatic aortic stenosis in patients with heart failure and aortic regurgitation contribute to a greater demand for this treatment method. The development of TAVI, as well as new designs of transcatheter valves, stimulate an ever deeper study of the anatomical and functional characteristics of the aortic root [3] since the success of any surgical intervention is based on a keen understanding of the anatomical features of structural pathology.

The term “aortic root” refers to the part of the ascending thoracic aorta, which is limited basally by the plane of the annulus fibrosus, cranially by the plane of the sinotubular junction [4]. The aortic root includes three main anatomical components: the valve, the sinuses of Valsalva, and intervalvular triangles, and, according to some authors, coronary ostia [5]. The aortic root lies between the left ventricle and the aorta. Its main function is to balance the pressure in the left ventricle and in the aorta.

In the case of TAVI, an incorrect assessment of the aortic root structure at the stage of preoperative imaging and, accordingly, an incorrect choice of the parameters of the implanted bioprosthesis entail intervention failure and/or postoperative complications. Thus, if the size of the selected prosthesis is smaller than optimal, this leads to paravalvular regurgitation or prosthesis migration, while oversizing is fraught with rupture of the annulus fibrosus, incomplete opening of the prosthesis with a subsequent risk of central transvalvular regurgitation, as well as conduction disturbances due to excessive pressure on the cardiac conduction system in the left ventricular outflow tract [6].

A standardized technique for preoperative computed tomography scanning and interpreting the obtained images with performing certain measurements has been developed [7]. However, currently, insurmountable obstacles persist in the form of the impossibility of assessing the functional component of the pathology on the basis of computed tomography, and a high risk of such a formidable postoperative complication as transverse atrioventricular blockade still remains. Thus, an update on diagnostic procedures is required in order to implement a personalized approach.

**The aim of the study** was to identify new anatomical landmarks of the aortic root and the association between the sizes of anatomical structures using the method of computed tomography angiography to improve models of heart valves and the methods for their selection in clinical practice.

## Materials and Methods

The dataset of computed tomography angiography (CTA) of 262 patients with aortic valve stenosis, including 99 men (37.8±3.0%) and 163 women (62.2±3.0%), were retrospectively analyzed. The mean age was 75.0±5.9 years. Severe aortic valve calcification was observed in 140 patients (53.4±3.1%), moderate calcification was found in 72 patients (27.5±2.7%), mild calcification in 30 patients (11.5±2.0%), no aortic valve calcification was found in 20 patients (7.6±1.6%).

The patients were divided into two groups:

group 1 consisted of 251 patients (95.8±1.2%), including 159 women and 92 men, with tricuspid aortic valve (TAV), their mean age was 75.0±5.9 years;

group 2 consisted of 11 patients (4.2±1.2%), including 7 men and 4 women, with bicuspid aortic valve (BAV), their mean age was 69.9±6.5 years.

The patients of the TAV group were subjected to a detailed analysis.

As a preoperative preparation for TAVI, all the patients underwent CTA on a Toshiba Aquilion One 320-slice computed tomography scanner (Toshiba, USA) with a reconstructed slice thickness of 0.5–1.0 mm. The scanning area extended from the apex of the lungs to the lower edge of the acetabulum to include the proximal brachiocephalic arteries (cranially) and the proximal iliac arteries (distally) into the study area. The rate of the contrast agent administration was 4.5–5.0 ml/s. The volume of the contrast agent injection was calculated by the formula:

*V* = injection rate **·** total scan time.

The analysis of the acquired images and all the measurements were performed on a specialized workstation by three radiologists with 5 or more years of experience.

**Figure 1.**
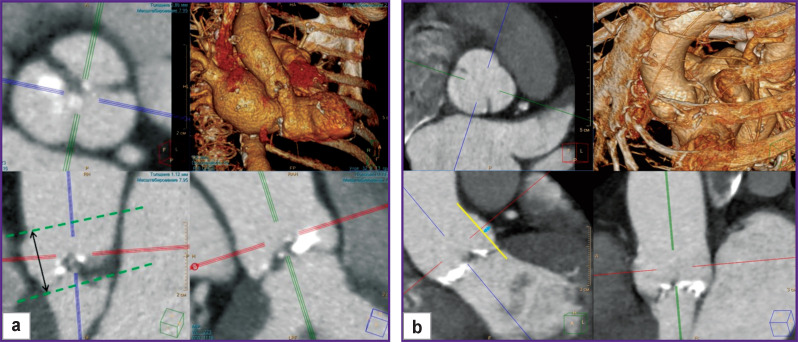
MPR (multiplanar reformation) reconstruction of the aortic root area: (a) measurement of the height of the sinuses of Valsalva: the green dotted lines indicate the planes of the sinotubular junction and the annulus fibrosus, the black arrow is the distance measured between them (the height of the sinus); (b) measurement of the depth of the sinuses of Valsalva: the yellow line passes through the points of the circle of the sinotubular junction and the annulus fibrosus, the blue arrow indicates the depth of the sinus of Valsalva

The measurements of the annulus fibrosus, sinotubular junction, and the height of the sinuses of Valsalva were performed according to the guidelines on measuring the aortic root for planning aortic valve replacement [7]. The aortic diameter at the level of the annulus fibrosus and sinotubular junction was calculated as the average between the maximum and minimum measurements. The mean height of the sinuses of Valsalva was calculated as the average of three segments drawn from the plane of the annulus fibrosus to the plane of the sinotubular junction through the center of the sinus semi-arch (Figure 1 (a)).

The scheme for measuring the depth of all the three sinuses of Valsalva is shown in Figure 1 (b).

The type of aortic root was determined by the formula:

K=hD.

where *h* was the mean height for a given patient from the annulus fibrosus to the sinotubular junction (mm); *D* was the mean diameter of the annulus fibrosus for a given patient (mm). The *K* coefficient was calculated for each patient.

### Statistical methods

The statistical analysis was performed using SPSS Statistics 24.0 software for Windows (SPSS Inc., USA). The normality of the distribution of continuous variables in each group was checked using the Shapiro–Wilk test. Since the distribution was normal, the results were expressed as mean and standard deviation M±σ. The significance of differences was evaluated by the Student’s t-test, considering the differences to be statistically significant at p<0.05. The Pearson correlation coefficient (r) was used to assess the strength of association between the compared measurements. The body mass index was calculated using the Du Bois and Du Bois formula [8]. The unpaired Student’s t-test was used to compare the continuous variables between the groups. A p<0.05 value was considered statistically significant.

## Results

### The tricuspid aortic valve group

**Figure 2.**
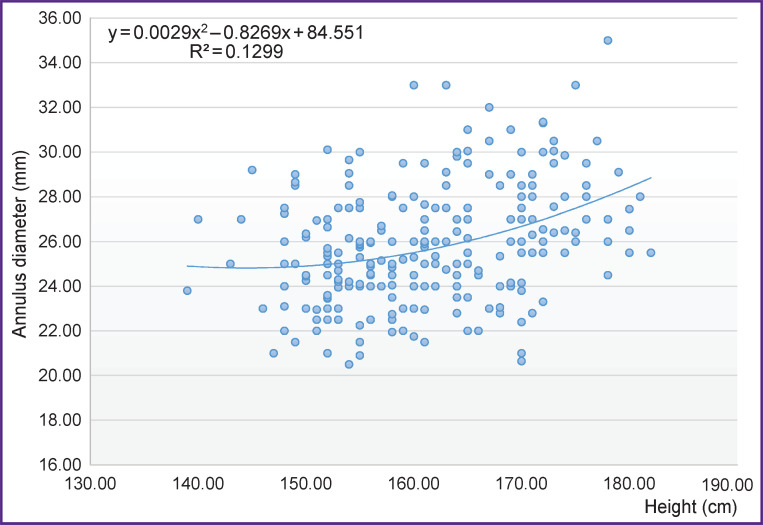
Correlation between the diameter of the annulus fibrosus and human height

**Figure 3.**
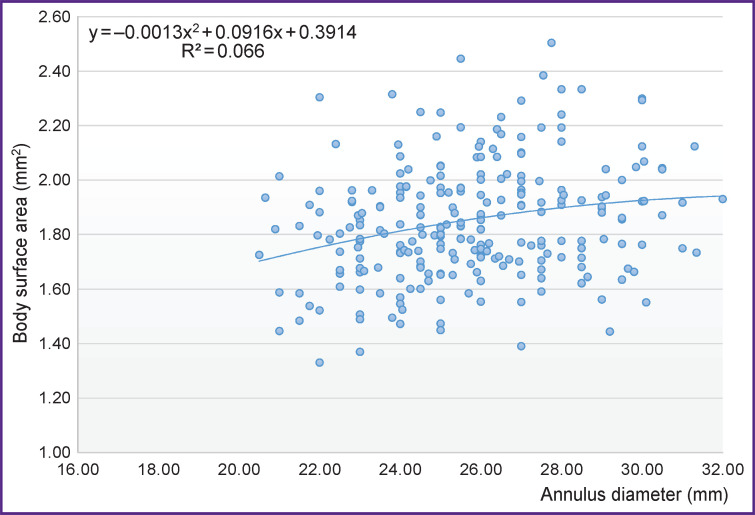
Relationship between body surface area and annulus diameter

In more than 50% of the cases, the diameter of the annulus fibrosus in the patients of the TAV group ranged from 23 to 26 mm (Table 1). At the same time, we did not find significant association between the diameter of the annulus fibrosus and patient’s height (r=0.35; p=0.01), or body surface area (r=0.25; p=0.01) and the height of the sinuses of Valsalva (r=0.34; p=0.01) (Figures 2 and 3).

**Table 1:** Diameter of the annulus fibrosus and the height of the sinus of Valsalva in patients with a tricuspid valve (n=251)

Diameter (mm)	Number of patients		Mean value of the sinus of Valsalva (mm), М±σ
	n	P±σ_P_ (%)	
21–22	19	8.0±1.7	18.5±1.7
23–24	56	22.0±2.6	18.2±1.6
25–26	83	33.0±3.0	19.2±2.1
27–28	48	19.0±2.5	20.1±2.4
29 and more	45	18.0±2.4	20.8±2.3

It is worth noting the presence of an average strength of association between the diameter of the annulus fibrosus and the diameter of the sinotubular junction (r=0.56; p=0.01).

**Figure 4.**
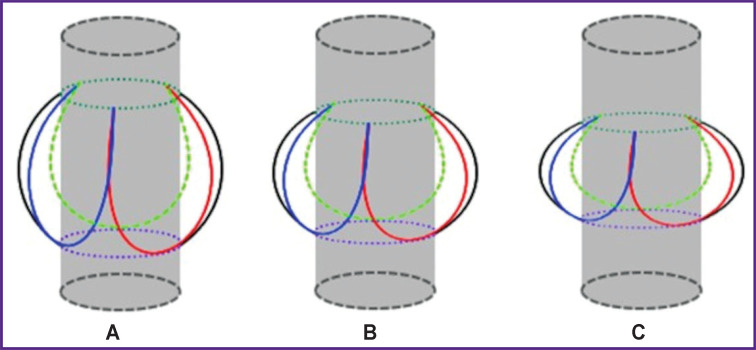
Schematic representation of types A, B, and C of the aortic root

When analyzing CTA data based on measuring the height of the sinuses of Valsalva and the diameter of the annulus fibrosus, three variants of the aortic root structure were identified: type A — *K*>1.05; type B — 0.95≤*K*≤1.05; type C — *K*<0.95 (Figure 4).

In 1 of 251 patients (0.4%), the *K* coefficient was greater than 1.05; in 4 of 251 patients (1.6%), it ranged from 0.95 to 1.05, which corresponds to types A and B of the aortic root, respectively. In the other cases (n=246; 98.0±0.9%), type C of the aortic root prevailed. At the same time, in 63 patients (25.1±2.7%), the *K* coefficient ranged from 0.80 to 0.89; in 99 patients (39.4±3.1%) — from 0.70 to 0.79; in 50 patients (19.9±2.5%) — from 0.60 to 0.69, which in total is 84.5±2.3% of the total number of patients with the type C aortic root (Table 2).

**Table 2:** Distribution of patients into groups depending on the annulus diameter, n (%), and calculated *K* (n=251)

***К* coefficient**	**Diameter of the annulus fibrosus (mm)**					
	21–22	23–24	25–26	27–28	29 and more	Group in total
**1.05–1.10**	1 (5)	0	0	0	0	1 (0.4)
**0.95–1.04**	2 (11)	2 (4)	0	0	0	4 (1.6)
**0.90–0.94**	3 (6)	2 (4)	8 (10)	2 (4)	0	15 (6.0)
**0.80–0.89**	6 (32)	16 (29)	20 (24)	15 (31)	6 (13)	63 (25.1)
**0.70–0.79**	7 (37)	25 (45)	30 (36)	15 (31)	22 (49)	99 (39.4)
**0.60–0.69**	0	9 (16)	22 (27)	10 (21)	9 (20)	50 (19.9)
**0.50–0.59**	0	2 (4)	3 (4)	5 (10)	8 (18)	18 (7.2)
**0.40–0.49**	0	0	0	1 (2)	0	1 (0.4)

The mean depth of the non-coronary sinus was 4.97± 0.97 mm, the depth of the right coronary sinus was 4.26±0.77 mm, the depth of the left coronary sinus was 4.60±0.81 mm, and the average for all the three sinuses was 4.61±0.71 mm. There was also no correlation between the diameter of the annulus fibrosus and the mean depth of the sinuses (r=0.31; p=0.01), however, a moderate degree of association was found between the depth and height of the sinuses (r=0.65; p=0.01).

### The bicuspid aortic valve group

In 2 out of 11 patients (18%), the preservation of all three sinuses of Valsalva was observed with fusion of two leaflets, while in 9 of 11 patients (82%), the differentiation of the right and non-coronary sinuses was difficult, while dilatation of the left sinus was noted, which was visually perceived as two symmetrical sinuses.

In this group, the most common diameter of the annulus fibrosus was 29 mm or more (4 patients, i.e., 36%), in 2 patients (18%) the diameter of the annulus fibrosus was 27–28 mm, in 1 patient (9%) it was 26–27 mm, also in 1 patient (9%) it was 25–26 mm, in 2 patients (18%) it was 23–24 mm, and in 1 more patient (9%) it was 22–23 mm.

2 patients (18%) in the BAV group had a type A of the aortic root, 1 patient (9%) had a type B, and 8 people (72%) had a type C

Given a small sample size of the bicuspid aortic valve group, no in-depth statistical analysis was performed.

## Discussion

Throughout life, the aortic valve is constantly subjected to repetitive mechanical stress, which increases with time due to arterial hypertension, which is very common in the population. As a result, sclerosis of the valve leaflets and their calcification develop [9]. As the disease progresses, violations of the mechanical function of the valve increase, those of stenosis or regurgitation. In addition, it has been established that a variant anatomy in the form of a bicuspid form of the aortic valve leads to its degeneration at an earlier age [10]. In our study, a similar result was obtained: in the BAV group, the mean age was 69.9±6.5 years; in the TAV group, it was 75.0±5.9 years.

In spite of the fact that studies on the features of planning the surgical treatment of aortic stenosis in patients with BAV prevail in the literature of the last decade [11, 12], our work focuses on TAV since this is the most common anatomical variant, and the incidence of postoperative complications and unsatisfactory treatment outcomes remains quite high in this category of patients, too [13]. This necessitates further improvement of diagnostic and therapeutic technologies, as well as medical devices under development.

Modern imaging techniques facilitate studying in detail the structural changes in the aortic root when planning surgery. To study the anatomical and functional parameters of the aortic root, it is possible to use diagnostic ultrasound, computed tomography, or magnetic resonance imaging. Each of these techniques has its own advantages and disadvantages. In our work, we used the findings on CTA as the most accurate method for sizing. CT is known to surpass many imaging methods in resolution, but it does not assess the functional viability of the elements of the cardiovascular system, since scanning with cardiosynchronization with data collection only in the diastolic phase is used to reduce radiation exposure. The literature indicates an increasing role of ultrasonic diagnostics in planning the surgical treatment of aortic stenosis [14]. And using three-dimensional ultrasound can minimize the main shortcoming of ultrasonic diagnostics — high operator dependence [15]. In the future, magnetic resonance imaging may become the most promising method for assessing the anatomical and functional characteristics of the aortic root and heart [16, 17].

In the present work, the diameter of the annulus fibrosus of the aortic valve was chosen as a key parameter since it is this that all standard size ranges and labeling of prosthetic heart valves are focused on. According to the obtained data, the most common diameter of the annulus fibrosus is 23–26 mm in patients with aortic stenosis. In healthy people, this diameter predominantly ranges from 20 to 22 mm [9]. We believe that aortic stenosis and valvular insufficiency naturally lead to an isolated enlargement of the annulus fibrosus.

This study has analyzed possible correlations between the size of the elements of the aortic root and general anatomical parameters of patients. No correlation between them and the diameter of the annulus fibrosus has been revealed. It should be noted that some sources report the presence of such association in healthy people [9].

Average correlation was found between the diameter of the annulus fibrosus and the diameter of the sinotubular junction, as well as between the depth and height of the sinuses of Valsalva. The first relationship can be explained by the interrelationship between the degree of stenosis of the aortic clan and pressure in the ascending aorta. It is believed that the greater the narrowing of the annulus fibrosus, the lower the pressure in the aorta is, since the volume of cardiac output decreases due to the obstruction of blood flow [18]. The correlation between the depth and height of the sinuses of Valsalva is probably anatomical and does not change considerably with the development of valvular pathology. In the present study, comparisons with the unchanged valve were not made, and we did not manage to find studies on correlation between the sizes of the sinuses of Valsalva in patients without valvular pathology.

We have been the first to propose a classification of the aortic root depending on the ratio of the height of the sinuses of Valsalva to the diameter of the annulus fibrosus. Three types of aortic root have been identified, the largest group being the patients with type C (84.5±2.3%), in whom the diameter of the annulus fibrosus prevails over the height of the sinuses, and the proposed *K* coefficient ranges from 0.60 to 0.89.

On the whole, the obtained findings indicate the individuality of the structure of the aortic root, which is not associated with other general and local anatomical parameters. All this indicates the need for further development and implementation in clinical practice of high-tech methods, the mathematical modeling of the aortic root for each patient with the possibility of virtual selection of the optimal transcatheter bioprosthesis model in particular.

The study of variants of the aortic root structure, the identification of relationships between the structures of the root allows for a more accurate assessment of its configuration. These data are necessary for those researchers who develop new models of transcatheter valves, the use of which will reduce the number of complications in the postoperative period. It should be taken into account that the C type of the aortic root is most common, and the wider the aortic root, the lower the height of the sinuses of Valsalva in relation to the diameter of the annulus fibrosus is; respectively, implanting a bioprosthesis carries a risk of overlapping the coronary ostia. On the other hand, if a valve is used with attachment in the ascending aorta, then with a decrease in the height of the implantable valve, the area of the landing zone decreases, which may cause valve migration.

The data obtained in this study will not only allow researchers to broaden their understanding of the structural pathology of the root in aortic valve stenosis and help in the selection of the type of bioprosthesis, but will also help find new ways to solve many problems in the development of new models of transcatheter devices.

## Conclusion

Based on the use of a personalized approach to detail the features of the aortic root in aortic stenosis, a classification has been proposed which will be useful not only for practitioners when choosing a treatment method, but also for researchers in understanding the structural characteristics of the aortic root in patients with its pathology so as to improve and develop fundamentally new models of artificial valves.
